# Improved assembly of the *Leishmania panamensis* strain PSC-1 genome

**DOI:** 10.1128/mra.00627-25

**Published:** 2025-07-21

**Authors:** Aldair Fabrega, Ana Cristina Ortega, Letizia Pardo, Carlos M. Restrepo, Alejandro Llanes

**Affiliations:** 1 División de Salud Humana y Enfermedades, Instituto de Investigaciones Científicas y Servicios de Alta Tecnología AIP (INDICASAT-AIP) 380695 , Panama City, Panama; 2 Facultad de Ciencias de la Salud, Universidad Latina de Panamá 125801, Panama City, Panama; 3 Facultad de Ciencias y Tecnología, Universidad Tecnológica de Panamá (UTP) 252900, Panama City, Panama; 4 Sistema Nacional de Investigación (SNI), Panama City, Panama; University of California Riverside, Riverside, California, USA

**Keywords:** *Leishmania panamensis*, genome announcements, trypanosomatids, intracellular parasites

## Abstract

*Leishmania panamensis* is a causative agent of leishmaniasis in Latin America, with significant incidence in Panama and Colombia. The first annotated genome draft for this parasite remained incomplete due to its highly repetitive nature. Here, we combined short- and long-read sequencing data to improve this genome draft.

## ANNOUNCEMENT

A draft of the *Leishmania panamensis* strain PSC-1 genome, released in 2015 (1), remained the only annotated genome available for this species in the GenBank and RefSeq databases at the time of this study. This draft was assembled using 454 pyrosequencing and Illumina reads, resulting in several unfinished regions mainly due to tandem gene arrays. Such arrays arise from the amplification of specific genes, a mechanism allowing *Leishmania* and other trypanosomatid parasites to modulate gene dosage in absence of transcriptional regulation of gene expression (2). The abundance of gene arrays and other repetitive elements often leads to highly fragmented assemblies when sequencing trypanosomatid genomes (3). With the advent of long-read platforms, such as PacBio and Oxford Nanopore (ONT), several reference genomes have been re-assembled to solve issues related to fragmentation (4–6). Here, we used a combination of ONT and Illumina reads to improve the *L. panamensis* strain PSC-1 genome draft.

Strain MHOM/PA/94/PSC-1 was isolated in 1994 from a skin lesion of a human patient from Panama. Cryopreserved parasites were cultured in serum-supplemented Schneider’s insect medium incubated at 25°C. Genomic DNA was extracted using the Wizard Genomic DNA Purification Kit (Promega). The ONT library was prepared with the Ligation Sequencing Kit SQK-LSK110, without shearing or size selection. This library was sequenced using an R9.4.1 flow cell in a PromethION instrument, generating approximately 581,000 reads (*N*_50_ = 17 kb). Guppy v.3.2.4 (HAC model) and Porechop v.0.2.4 were used for base calling and adapter trimming, respectively. The Illumina library was prepared with the TruSeq DNA HT Sample Prep Kit and sequenced in a HiSeq 2000 instrument, yielding 29.4 million 100 bp paired-end reads. FastQC v.0.11.19 and Trimmomatic v.0.39 (7) were used for quality control and trimming, respectively. Canu v.2.2 (8) was used to assemble the ONT reads *de novo* with the “genomeSize=34m” option, resulting in a set of 35 non-redundant contigs (*N*_50_ = 1,281 kb), consistent with the 35 *L*. *panamensis* chromosomes. Illumina reads were then used for error correction with Pilon v.1.24 (9) in seven iterations. Illumina and ONT reads were aligned to the polished assembly with BWA-MEM v.0.7.17 (10) and Minimap2 v.2.24 (11), respectively. These alignments were visually inspected in Artemis v.17.0.1 (12) to detect possible misassemblies in regions that were not supported by the reads, all of which were manually corrected. Default parameters for each type of read were used for all software except otherwise noted.

Annotation of the improved assembly was performed with RATT v.1.0 (13) and the Companion server v.2.2.11 (14) to transfer previously annotated genes and to identify new ones, respectively. When compared with the 2015 genome draft, the improved assembly contains more than 3 Mb of additional sequence (Table 1). Newly assembled regions putatively encode more than 800 protein-coding genes, many of which were confirmed to be located within tandem gene arrays. Assessment of genome completeness with BUSCO v.5.4.4 (15) identified 100% of the genes in the “euglenozoa_odb10” lineage data set. The presence of *Leishmania* telomeric repeats (16, 17) was also confirmed near chromosome ends.

**TABLE 1:** Global statistics of the first two versions of the *Leishmania panamensis* strain PSC-1 genome

Genome statistic	Version 1 (2015)^*a*^	Version 2 (2025)
Total size (Mb)	30.7	33.9
Sequencing coverage	100×	100×
Number of chromosomes	35	35
Chromosome length range (Mb)	0.3–2.6	0.3–3.1
GC content (%)	57.6	58.2
Number of gaps	553	0
N content (%)	2.3	0
Protein-coding genes	7,748	8,621
Pseudogenes	185	219

^*a*^ Data taken from Llanes et al. (1), provided here only for comparative purposes.

## Data Availability

The sequence reads, assembly, and annotation reported here are available in GenBank under BioProject PRJNA235344. Raw sequence reads have been deposited in the Sequence Read Archive (SRA) under accession numbers SRX681913 for Illumina reads and SRX28833427 for ONT reads. Annotated chromosomes were deposited in GenBank with accession numbers CP009370.2 to CP009404.2. The version described in this paper is the second version.
